# Boundary Objects: Engaging and Bridging Needs of People in Participatory Research by Arts-Based Methods

**DOI:** 10.3390/ijerph18157903

**Published:** 2021-07-26

**Authors:** Barbara Groot, Tineke Abma

**Affiliations:** 1 Amsterdam University Medical Centre, VU Medical Centre, Department Ethics, Law and Medical Humanities, De Boelelaan 1089a, 1081 HV Amsterdam, The Netherlands; t.a.abma@lumc.nl; 2 Leyden Academy, Rijnsburgerweg 10, 2333 AA Leiden, The Netherlands; 3 Leiden University Medical Centre, Leiden University, Albinusdreef 2, 2333 ZA Leiden, The Netherlands

**Keywords:** boundary objects, participatory health research (PHR), arts-based methods

## Abstract

Background: Participatory health research (PHR) is a research approach in which people, including hidden populations, share lived experiences about health inequities to improve their situation through collective action. Boundary objects are produced, using arts-based methods, to be heard by stakeholders. These can bring about dialogue, connection, and involvement in a mission for social justice. This study aims to gain insight into the value and ethical issues of boundary objects that address health inequalities. A qualitative evaluation is conducted on three different boundary objects, created in different participatory studies with marginalized populations (mothers in poverty, psychiatric patients, and unemployed people). A successful boundary object evokes emotions among those who created the objects and those encountering these objects. Such objects move people and create an impulse for change. The more provocative the object, the more people feel triggered to foster change. Boundary objects may cross personal boundaries and could provoke feelings of discomfort and ignorance. Therefore, it is necessary to pay attention to ethics work. Boundary objects that are made by people from hidden populations may spur actions and create influence by improving the understanding of the needs of hidden populations. A dialogue about these needs is an essential step towards social justice.

## 1. Introduction

Participatory health research (PHR) [1,2] is a research approach in which people, including those who appear marginalized concerning the academic system, conduct a study together with academics and other stakeholders to make a difference in their lives. The people from the so-called ‘hidden populations’ are co-researchers with a lived experience. We write hidden populations within quotations because being hidden is not a characteristic of people, but of our social relations that create invisibility. People without a face and voice collaborate with academic co-researchers in PHR, who often facilitate the process of the study. In addition, PHR aims to learn about important topics in the lives or work of co-researchers. Social justice is a core principle of PHR. The lessons learned in the first phase of a PHR project must be shared with other stakeholders to generate collaborative strategies for change. The idea is that stakeholders, who often are embedded in a system of (governmental) institutions, are engaged to make a difference and reduce health disparities. Some stakeholders are not ‘hidden,’ but are highly visible due to their role and function in organizations.

The first phase of a PHR study often focuses on sharing and reflecting on lived experiences [3]. However, sharing lived experiences is not always easy for people, especially for people in poverty, those who are marginalized, etc. They often feel shame in expressing their experiences and want to hide from the gaze [4] of society. Their experiences often relate to painful or harmful moments and are often messy stories. The lived experiences are often bodily experiences and the painful feelings are hard to share, and challenging to articulate with words [5,6]. Arts-based methods help uncover these experiences in another form of knowledge, namely presentational knowledge [7]. Presentational knowledge emerges from experiential knowledge, and provides the first form of expressing meaning and significance by drawing on expressive forms of imagery through dance movement, music, drawing, painting, sculpture, poetry, story, drama, and others. Presentational knowledge visualizes the knowledge that is difficult to express in words. 

In the second phase of a PHR study, encounters and dialogue occur with other stakeholders after studying the lived experiences [3]. These stakeholders are often not (totally) aware of the lived experiences of the hidden populations. By sharing the arts-based products made during PHR, which transfers the bodily experience of the lived experience to others, the PHR research team attempts to connect with others and evoke empathy. The aim is to heighten the relevance of the lived experiences, and find future partnerships for positive change and social justice by reducing health inequities with (versus for) the people concerned through a mutually transformative power relationship [8,9].

When the creative output of participatory arts-based methods begins to travel through various worlds and evokes action, we speak of ‘boundary objects’ [10,11]. A boundary object denotes a concept of objects that inhabit several intersecting social worlds. These objects satisfy the communicative and performative needs of each of them. Star and Griesemer elaborated as follows:

*Boundary objects are objects which are both plastic enough to adapt to local needs and the constraints of the several parties employing them, yet robust enough to maintain a common identity across sites. They are weakly structured in common use, and become strongly structured in individual site use. These objects may be abstract or concrete. They have different meanings in different social worlds, but their structure is common enough in more than one world to make them recognizable, used as a means of translation. The creation and management of boundary objects is a key process in developing and maintaining coherence across intersecting social worlds*.

([11], p. 393)

Since 1989, the concept of the boundary object has traveled to many fields; for example, from museums to international development, agriculture, and management. In addition, in health care, boundary objects are used to mitigate language and knowledge boundaries between different disciplines [12].

A boundary object need not be a material object, but can also be an artifact, practice, representation, or technology [11]. Boundary objects could be anything [13] from budgets, timelines, targets [14], maps, and visual representations [11], to such concepts as ‘resilience’ [15], ‘transformation’ [16], or ‘participation’ [13]. Boundary objects could enable different stakeholders to temporarily align themselves around a common project for a common purpose. Thus, even a concept or idea, such as ‘hidden populations’ or ‘health equity,’ could be a boundary object. In PHR, the connections between various worlds and mutual learning are central. Boundary objects may fulfill these needs, and many boundary objects have been created within PHR to date; however, these are often not presented as such in the literature, for example comics [17], canvases [18], video vignettes [19], and narrative or visual metaphors [20]. These boundary objects are often material artworks, collaboratively made by participants living in marginalized situations, whose faces and voices are unseen and unknown. The objects are often displayed in public spaces or sessions to exchange knowledge with stakeholders, and create empathy about experiential knowledge.

Although the value of participatory approaches in public health to address health inequities and the pathways of arts-based methods are discussed in the literature [1,21,22], little is known about the pathways of boundary objects in addressing the health inequities of people from hidden populations. Various scholars have tried to pinpoint the working pathways of boundary objects. Table 1 presents an inventory of pathways from the literature. These are all in the field at the professional or educational level, not from people in marginalized situations.

Moreover, from practice, we experienced that working with boundary objects is not always easy. As boundary objects allow nonhierarchical collaboration, there is a strong need for attention of the process of equal and genuine dialogue. A boundary object creates an arrangement for joint activity, but not without effort. It requires time for assembly, trust-building, contemplation, remembrance, and argument and dispute, alongside other forms of emotional and embodied engagement. However, in the literature, there is not enough attention focused on these prerequisites for successful boundary objects. Boundary objects could be dismissed if they fail to meet stakeholders’ requirements, and stakeholders can resist the objects. As far as we know, only one scholar [25] has provided an example from her work in arts and health. She indicates that artworks become weak boundary objects if, for example, the media focuses only on their aesthetics and value for the healthcare environments, instead of on the individual and social significance of these objects for patients and caregivers. Stakeholders can easily perceive artworks as diverting resources away from patient care ([25], p. 15).

This study aims to present the value and pathways of boundary objects as part of PHR to address health inequalities and prerequisites for ethical collaboration with people in marginalized situations. This article presents three examples of boundary objects made and used for dialogue sessions in PHR. We evaluate these objects on their value and pathways, using the lens of the pathways listed in Table 1. Moreover, we discuss the ethical challenges that occurred in the dialogue sessions. With this study, we hope to enrich the understanding and the possibilities, through exploring the needs of hidden populations and their role in increasing visibility, strengths and capacities.

## 2. Materials and Methods 

The method in this study is an evaluation of three boundary objects from different PHR studies. The authors used purposeful selection [26] in this study. Three PHR studies with various groups in different contexts were selected, using three different boundary objects with different art forms (spoken word, a report with a song, and an illustrated narrative). All three objects were cases with learning potential; thus, although these objects are not representative, they provide a better understanding of the pathways that boundary objects may fulfill for hidden populations to conquer health disparities, in the context of PHR [27]. Focusing on objects with learning potential helps to illustrate matters overlooked in typical cases [28]. The selection was immediately apparent for the authors because these objects stood out and were thought-provoking, revealing their complexity related to ethical issues and social dynamics. All boundary objects affected the PHR studies and beyond. Therefore, the authors felt an urgency to understand the pathways, in order to learn from them. Table 2 summarizes the background in which the boundary objects were created and used.

First, the authors described their case from their perspective. Both B.G. and T.A. were involved in the three cases as participatory researchers and were involved in constructing and disseminating the boundary objects. In addition, B.G. analyzed the data from the PHR projects, especially the data around the moment that we disseminated or used the boundary objects. The data consisted of audiotapes, videos, transcripts, diary notes, and reports. All PHR studies were conducted from 2016 to 2020 [29]. Secondly, we analyzed the data using the frameworks described in Table 1.

## 3. Results

In this section we present the three boundary objects and share our analysis per object.

### 3.1. DebTalk

**Figure 1:**
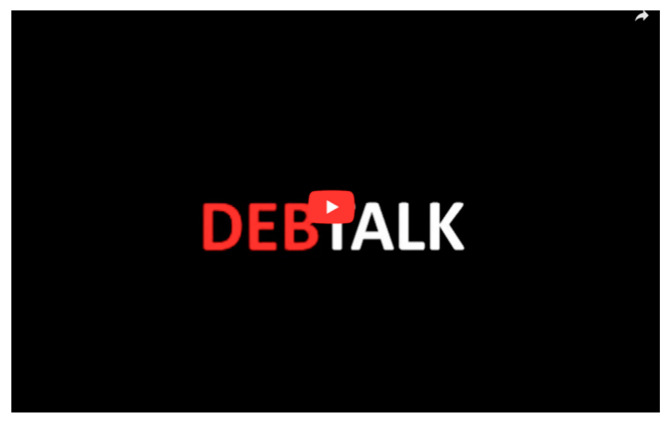
A video with a monologue, which was also performance in a theater in town, here: https://youtube/AyEVUhUHyXE (accessed on 25 July 2021).

In a study about the service delivery of the municipality, a group of people who were unemployed created different artworks about their experiences of being poor and unemployed [30,31]. One of them created a performance with a monologue. In this performance, Debbie was behind the scenes and read her story out loud. At the end of the story, she presented herself and finished her monologue: “I am Debbie, I am a human being”. Debbie also created a video of this monologue (Figure 1) to use in other settings. As a group, we presented the artworks three times to a different audience of stakeholders who could effect change. After the presentation, we asked the audience to reflect on the objects and write down their thoughts. The stakeholder’s reflections focused on the complexity of the problems Debbie encountered in her life, and the need to ‘see’ all citizens as human beings.

*“What wonderful, powerful presentations. But complex and intricate. The presentation touched me. They were moving. How much we learn from this meeting. The most important thing is to ‘really’ see each other, to be attentively curious about each other, person to person.”*

Two of the three performances were in a small safe setting, in which the group of people without a job felt safe and invited outsiders to listen to them. The final performance was held on a festival stage with an unknown audience of 180 people. After the presentation, the moderator asked for some general reflections. One person felt responsible for reacting from the municipality’s position. In a plenary discussion, Debbie, her group, and the municipality representative began to argue. Both parties felt attacked and felt the need to bring their arguments to the forefront. An ethical plenary dialogue about the pain that people without a job could feel was not possible in this setting. The conversation’s focus was on the “failure of service” and “defense of the municipality’s working forces.” It asked for of the facilitators and moderator to operate in an atmosphere of safety and dialogue.

### 3.2. Make Contact with Me

**Figure 2:**
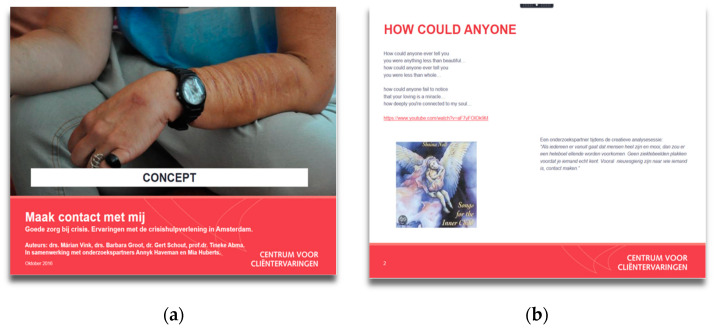
(**a**) A report with photographs and title: “Make Contact with Me” (**b**) the first page of the report with the song text “How Could Anyone”, see https://www.youtube.com/watch?v=aF7yFOlOk9M (accessed on 25 July 2021).

In another PHR study about emergency psychiatric care, a group of researchers and people with lived experiences organized a dialogue with people who worked in psychiatric emergency care [32,33,34]. One of the main findings was reported in “Make Contact with Me.” The co-researchers with lived experiences chose to present the results in a report with a visual of a client’s arm on the cover (Figure 2). In order to share the clients’ feelings in emergency care with professionals who work in this field, we started the dialogue sessions with a short introduction by a co-researcher. We played the song “How Could Anyone” by Shiana Noll. This song represented the feeling of clients not being ‘seen.’ The colleague with lived experience introduced the theme:

*“If everyone assumes that people are whole and beautiful, a lot of misery would be avoided. No pasting labels before you really know someone. Especially being curious about who someone is, making contact.”*

The participants in the dialogue session were silent after the song. In informal conversations, we heard that some understood the song’s message but felt attacked. They felt that the object was focused on the way they did their jobs: this was not correct. They felt as if they failed their duty—a duty they loved, and it invalidated why they started to work in psychiatric care. It was a confrontational message, and not everyone was ready to reflect on this message. The atmosphere was one of disruption and resistance. It was not easy to enter into a dialogue about the findings because people felt they had to defend their practice.

In contrast, some professionals indicated that they were happy that this message was conveyed. It was precisely the way that they saw the situations and felt they were a victim of the system. They embraced the findings of the study to make a change in their organization. 

### 3.3. You Do Not See It

**Figure 3:**
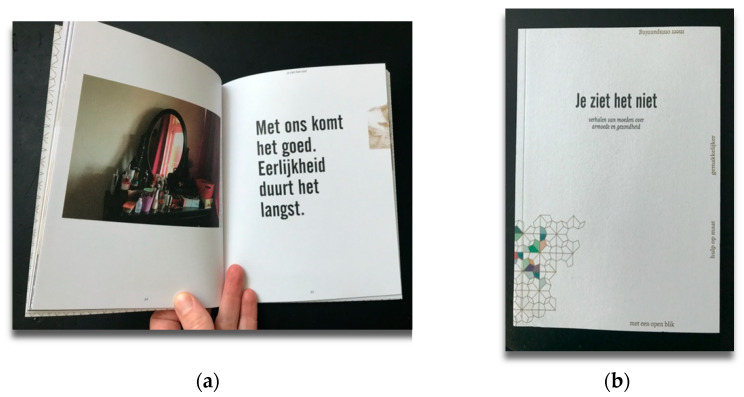
(**a**) One of the pages in the booklet with a photo made by a co-researchers, and a quote from her “We will be fine. Honesty is the best policy.” (**b**) The cover of the booklet with the title ‘You Do Not See It’: https://www.fnozorgvoorkansen.nl/wp-content/uploads/2019/12/Jeziethetniet_FNO-002.pdf (accessed on 25 July 2021).

A group of mothers living in poverty conducted a PHR study for four years [35]. They worked with a funding agency to improve the health promotion projects in neighborhoods, focusing on families with a low socioeconomic status. In this study, as a team, we made a booklet about their lives (Figure 3). They wanted to show the world how they lived their lives and what was essential for them. They wrote about the stress they experienced in dealing with authorities, and about being seen as an untrustworthy person. They also wanted to clarify that the focus needs to be on reducing stress, rather than reducing weight or alcohol use to improve their health. The photographs bring the audience behind the backdoor of the mothers, and allow them to visually illustrate their stories. The readers encounter imagery that is not always expected. 

As a group, the mothers and researchers organized 10 meetings with policymakers, professionals in care and well-being, and people who help with unemployment. In these meetings, we asked professionals to read the booklet beforehand. We presented a few parts of the brochure in spoken word form, on topics that the audience wanted to reflect on. We also invited the audience to bring in questions and dilemmas regarding their practices to discuss with the mothers. 

A focus on the dialogue, questions, and dilemmas of the professionals was new to us. In other projects, we only shared the objects of the hidden populations. The booklet was a way to connect, but the focus was on deeper topics of conversation. In summary, the focus was not solely on sharing stories of people from a hidden population. The makers (mothers living in poverty), social designer, and the funding agency collaboratively decided on this because they wanted more than to share the hidden stories.

*“This conversation made it clear again that sometimes we are not on the right route... Excellent feedback, professional, judgment-free, and positively worded yet not misunderstood. It has put us over the top to change things.”*

*“Clarifying. It gave me confidence that we are doing a good job in the schools with the children and that that is enough for now. It doesn’t always have to be even grander and more; sometimes we just want too much and too fast.”*

*“Very nice to talk with this group about our project. They looked at it from a different perspective, and that gave interesting insights. Through such a conversation, you focus consciously on the target group and get more understanding.”*

In this study, it seemed that no ethical issues from the dissemination of the boundary object occurred. A more traditional, less provocative medium, such as a booklet with narratives and photographs seemed to neutralize feelings. This object is less multisensory than other objects with voice, music, or performance. It is also an object that is more acceptable in a policy environment. Moreover, the booklet was more powerful in its form because it was designed in the corporate identity of the funding agency. However, as the format made it possible to be comfortable with boundary objects that were not threatening to the stakeholders, some parts of the experience were not visible or felt by the audience, which could also be considered as a major ethical issue.

## 4. Discussion

This study focuses on boundary objects, which intend to uncover hidden populations. Thus, the concept of a boundary object defines the existence of two worlds: the ‘hidden’ world and the ‘not hidden’ world. Moreover, PHR focuses on connecting the people in these two worlds, aiming for social justice through ensuring the visibility of all. We found that boundary objects could help to bring both worlds together. However, boundary objects can cross borders, and can often evoke uncomfortable experiences of these borders. Boundary objects create an arrangement for joint activities, but not without effort. They require work to be completed by PHR researchers, who facilitate the process of border crossing, and effort from the hidden population and audiences. 

Firstly, we reflect on the work that people from hidden populations must perform in the process of making a boundary object. They must often cross a threshold to overcome shame or pain, in order to make their experiences visible, and bring them out in the open. In doing so, they hope that stakeholders, who can effect change and reduce health disparities and broader social inequities, will address their needs. Expressing these needs and emotions is often limited by language and cognition. These experiences are often unsayable [36]. Creative methods help to express these experiences, but it remains a laborious process to expose themselves. People are often disappointed by agencies and professionals, and have little faith that policymakers or others will listen to their stories and needs. In practice, not everybody was as interested in the object and stories as we hoped. 

Secondly, the audience must also work to relate to the boundary object. The audience must see, feel, and hear the message of the hidden population. They are confronted with something different from another world. This confrontation with this strange world, which cannot be articulated in words, can be difficult. Sometimes this confrontation creates small changes, including the example of the DebTalk, where we were in a small group in which everybody felt safe. Policymakers could show something of their own, so they were open to discussion. This exchange made the setting more equal from person to person (not from client to professional). Both shared personal perspectives, which equalized the power hierarchy. In the example of psychiatric care, the confrontation was too uncomfortable; it did not generate an open conversation about the underlying emotions and experiences at that particular moment. The different, hidden and unhidden worlds met, but the boundary object reinforced differences and magnified stereotypical imagery and latent tensions, making power asymmetries more tangible. There was no willingness in the moment to settle or work across that boundary. The power relationship between patients and health care professionals remained intact, and the hidden population remained hidden. 

In summary, it takes work from both sides to meet and equalize power. This article reveals that ‘boundary spanners’ [37,38] are necessary for participatory research. These are not ‘brokers,’ a commonly applied term in the health sector to describe a person engaged in multiple functions. ‘Boundary spanners’ are people who engage in relationship building [39]. In the cases in this study, the researcher was a boundary spanner, and some co-researchers also took this role. For example, the mothers felt a responsibility in spanning boundaries, and their role and function in the context was focused on it. However, the psychiatrists were not able to span boundaries. Future studies would profit from strengthening a relational understanding of power bases, which would provide tools to detect the subtle workings of power in boundary-spanning processes. For example, expanding the notion of power bases to the demographic diversity of gender, race, or class. For example, when acts to adapt to boundary-spanning processes may be revealed as acts of micro-resistance [40].

Boundary objects are not always successful in crossing boundaries, especially in settings with a strong power hierarchy. For facilitators in these processes (participatory researchers), this requires ethics work [36] and work to create a safe and communicative space [29]. This type of social foundation for dialogue is necessary to meet ethical demands, to equip participants with the confidence to speak, willingness to share, and the sense that someone is listening to them [41]. The findings reminded us about the “pedagogy of discomfort” [42,43]. This pedagogy encourages people to leave their “comfort zones” and critically investigate the “Self” concerning the “Other”. In line with Fine [44], a pedagogy of discomfort calls for “working the hyphen” between the Self and Other:

*“Working the hyphen means creating occasions to discuss what is, and is not, “happening between,” within the negotiated relations of whose story is being told, why, to whom, with what interpretation, and whose story is shadowed, why, for whom, and with what consequence. …Self and Other are knottily entangled we inscribe the Other, strain to white our Self, and refuse to engage in contradictions [44].”*

She underscored how important it is to break out of sedimented identities, especially for people who find themselves locked up in the notions or labels placed upon them. The idea is that uncomfortable feelings challenge dominant beliefs, habits, and normative practices that sustain social inequities and Self-Other binaries. The discomfort and reflection create openings for change and transformation. 

In the third example, the group has partly consciously and unconsciously attempted to avoid the audience’s discomfort. The mothers dearly wanted a strategic partnership, rather than a connection with discomfort. The booklet of the mothers in poverty and the funding agency did not evoke feelings of discomfort, and did not set in motion an investigation of the self-concerning mothers from the hidden population. The book describes feelings that were neutralized in the object. The mothers from the ‘hidden’ population acted as if they were professionals engaging in the dialogue sessions about the booklet. The mothers wanted to be part of that “Other world” of professionals, such that they were willing to give up on their own identities. As a result, their own identity and the world they live in remains hidden. Again, the power of hierarchy plays a role in the dynamic that the boundary object sets in motion.

This article demonstrated how challenging it is to work with boundaries or across boundaries. An underlying question is: who draws the line or border between worlds that are separated? Boundaries are always about inclusion and exclusion. For example, hidden is not a characteristic of this group; however, we, as researchers or professionals, mark this group as such and thereby draw a line between them and us. We are complicit and construct boundaries that generate inclusion and exclusion. The hidden population only exists due to the unhidden or open population. The binary of hidden/unhidden is asymmetric, in which ‘unhidden’ or open seems to be better than ‘hidden’. The question is, who determines that? How does it work this way? In psychiatry, clients opened up but did not receive a positive response from their health care professionals. They tried to dissolve the boundary but were met with resistance, which generated the question regarding whether the openness about unhidden worlds is always good. Can there be toxic versus healing ways to uncover and cross boundaries?

In our studies, we deliberately created space for hidden populations, but whether we succeeded in this effort also depended on the openness of other stakeholders. In the project with the mothers, the hidden parts of their lives remained, to some extend, hidden because the context and power dynamics did not allow openness. All stakeholders in that context hid behind a mask of professional neutrality. In the workplace project, we saw an opposite dynamic. At the same time, some civil servants of the municipality were visible as professionals; they deliberately hid their personal lives and experiences with psychiatric and other vulnerabilities. Some parts of their selves could not be shared in the organizational context, but became visible during the dialogues with service users who dared to open up and show their vulnerabilities. In regard to who is and is not visible, and what is offered and hidden is thus highly relational and context-laden.

Finally, most arts-based PHR focuses on making boundary objects and sharing knowledge (step one from Melo and Bishop [12]). Creating a collective understanding, step two, is often a difficult step in these processes. The third step about solutions asks for much more. People in hidden worlds often experience complex situations that ask for long-term and structural commitments and solutions. We (and other stakeholders) could not solve these in the scope of a three year PHR project. In these studies, we focused on identifying a group in line with the theory by Akkerman and Bakker [24]. However, we could more appropriately call it “discomfort” and “reflection on the Self and Other”. We added these pathways in Table 3, next to the three other pathways from different backgrounds. 

## 5. Conclusions

Boundary objects made by people from ‘hidden populations’ may spur actions and create influence, by understanding hidden populations’ needs. A dialogue about these needs is an essential step towards change. The concept of discomfort to reflect on the Self and Other is a necessary start for transformation. It is vital to effect change. This process is not without any effort. People from hidden populations must work, particularly emotionally, to make objects that reflect their lived experiences. Participatory researchers must work on ethics, and create safe and communicative spaces to ensure that encounters provide an opening for transformation. Finally, the audience must work to encounter the Other, to reflect on the Self, and create occasions to discuss events within the relations of their stories. Discomfort is essential as a start. 

## Figures and Tables

**Table 1:** Three frameworks on the pathways of boundary objects from literature.

List of Pathways	Field	Authors
(1) knowledge sharing (2) collaborative meaning making (3) collective understanding and coming up with solutions	Healthcare (with professionals)	Melo and Bishop [12]
(1) identifying problem boundaries (2) orchestrating collective responsibilities (3) describe three similar organizing practices	Management studies	Hsiao, Tsai and Lee [23]
(1) identification (2) coordination (3) reflection (4) transformation	Learning theory	Akkerman and Bakker [24]

**Table 2:** Background of the boundary objects with learning potential in different PHR studies.

Title of Boundary Object	Format of the Objects	Creators of the Objects (Hidden Population)	Period of Creation	Background of Stakeholders	Setting of Sharing the Objects
DebTalk	Spoken word (on-/offline)	People without a job	2018	Service for employment	Theater and Festival of Participation
Make contact with me	Report with a song	People with psychiatric vulnerability	2018	Psychiatric emergency care	Psychiatric hospital
You do not see it	Narrative and photo story	Mothers in poverty	2019	Municipality, care, welfare	Different workshops

**Table 3:** Four frameworks on the pathways of boundary objects.

List of Pathways	Field	Authors
(1) knowledge sharing (2) collaborative meaning making (3) collective understanding and coming up with solutions	Healthcare (with professionals)	Melo and Bishop [12]
(1) identifying problem boundaries (2) orchestrating collective responsibilities (3) describe three similar organizing practices	Management studies	Hsiao, Tsai and Lee [23]
(1) identification (2) coordination (3) reflection (4) transformation	Learning theory	Akkerman and Bakker [24]
(1) sharing knowledge (2) discomfort (3) reflection on the Self and Other/working the hyphen (4) openings for transformation	Participatory health research	Groot and Abma
